# Rhazes, a Genius Physician in the Diagnosis and Treatment of Nocturnal Enuresis in Medical History

**DOI:** 10.5812/ircmj.5017

**Published:** 2013-08-05

**Authors:** Saeed Changizi Ashtiyani, Mohsen Shamsi, Ali Cyrus, Seyed Mohammad Tabatabayei

**Affiliations:** 1 Department of Physiology, Arak University of Medical Sciences, Arak, IR Iran; 2 Department of Public Health, Arak University of Medical Sciences, Arak, IR Iran; 3 Department of Urology, Arak University of Medical Sciences, Arak, IR Iran; 4 Medical Ethics and History of Medicine Research Center, Tehran, IR Iran

**Keywords:** Nocturnal Enuresis, Medical History, Rhazes

## Abstract

**Context:**

Nocturnal enuresis has undoubtedly occurred since man's earliest days and the first references are found in the Ebers papyri of 1550 BC. The purpose of this study is to review of Rhazes opinion about diagnosis and treatment of nocturnal enuresis and compare his belief and clinical methods with modern medical practice.

**Evidence Acquisition:**

In the review study we searched all available and reliable electronic and paper sources using appropriate keywords about the views of Rhazes, and compared them with recent medical evidence about diagnosis and treatment of nocturnal in medication.

**Results:**

Our findings proved that Rhazes described the symptoms, signs, and the treatment of nocturnal enuresis in accordance with contemporary medicine.

**Conclusions:**

A review of opinion Rhazes and other ancient Islamic medical textbooks on nocturnal enuresis reveals that medical practice in those days was comparable to modern medicine yet avoiding the side effects that are commonly experienced with the modern medical approach.

## 1. Context

Abu Bakr Muhammad Ibn Zakariya Rhazes was born in Rayy, a town on the southern slopes of Alborz Mountains near the present-day Tehran, Iran, in 865 AD. He was first interested in music but later studied chemistry, alchemy, and philosophy. Due to an eye irritation caused by the chemicals he was experimenting with, he was compelled to stop his experiments in alchemy at the age of 30 years. By then, he was credited for the discovery of sulfuric acid and ethanol. There is a debate between historians as to where and from whom Rhazes gained his medical knowledge, but both Jamal Al-din Al Ghefti and Ibn Abi Usaibia have mentioned that his teacher was Ali Ibn Rabban Tabari, which is almost certainly incorrect, as Tabari died at 861 AD (247 AH), while Rhazes was not born until 865 AD (251 AH). It is possible; however, that Rhazes had studied Tabari’s medical texts (such as Ferdows Al-Hakameh) and thus, he had referred to him as his teacher of medicine (1, 2).

Rhazes wrote more than 224 books on various subjects, but his most renowned manuscript was the medical encyclopedia, Al-Hawi fi al-Tibb, known in Europe as Liber Continens. It was a compilation of Greek and Roman medicine, his own clinical observations and case studies, and his personal medical practice. Rhazes extensively exploited case histories as an educational aid for the documentation of different diseases he diagnosed and treated. Rhazes pursued a comprehensive scientific methodology based on experiment which was in agreement with modern medicine and especially in the field of urology. For example urinary tract anatomy, addressed bladder and noctural enuresis (3).

Nocturnal enuresis is a very common condition and has undoubtedly occurred since man's earliest days and the first references may be found in the Ebers papyri of 1550 BC. (4). “Enuresis” is a term coined in 1790 and literally means “to urinate within” (5) and “nocturnal” means “occurring at night” ; thus, nocturnal enuresis is a medical term applied to what nowadays is called bed-wetting.

Nocturnal enuresis (NE) is the involuntary loss of urine in sleep in a child aged five years or more in the absence of congenital or acquired defects of the nervous system or the urinary tract. Most children usually achieve night-time dryness by the age of four or five but it is estimated that 20% of five-year-olds experience nocturnal enuresis. This affliction decreases with age and by the age of twelve, 3% of children still experience bed-wetting but after the age of 15, one percent of population continues to suffer from enuresis which persists into adulthood (5, 6). NE is more often seen in boys in the early years but equals out between both sexes in later years (7, 8). There is a familiar pattern in the transmission of bedwetting and some studies have proposed genetic inheritance as the culprit (7-10). Although NE is not accompanied by any serious physical morbidities, it can become a significant problem affecting children and their families and several studies have shown a deleterious effect on the self-esteem, behavior, development of social skills, and quality of life of those afflicted by it (11-13).

Enuresis is classified into primary and secondary types. Primary nocturnal enuresis (PNE) is the most common form and this term is applied to individuals who can control their voiding during the day but were not constantly dry at nights for at least a six-month period since their infancy. To make the diagnosis, the number of enuretic episodes should be at least twice a month in children 3 to 6 years of age and no less than one time per month in older subjects. A multitude of studies have been conducted on PNE but unfortunately, the cause of this disease is still elusive to contemporary medicine and in only 1 – 3% of cases an identifiable cause can be established (14). Secondary nocturnal enuresis (SNE) refers to recrudescence of bed-wetting after control has been achieved for at least six months. Twenty five percent of enuretic children have SNE and its prevalence as compared to PNE increases with age. Psychological factors play an important role in SNE but organic causes should be first ruled out in these patients (15).

One review found that when both parents were enuretic as children, their offspring had a 77 percent risk of having nocturnal enuresis. The risk declined to 43 percent when one parent was enuretic as a child, and to 15 percent when neither parent was enuretic (16). Another investigation found a positive family history in 65 to 85 percent of children with nocturnal enuresis (17).

Many studies on the results of the treatment of NE have been reported over the last ten years; however, the majority of the reports define “full response” as achieving a 90 percent reduction in wet nights (18, 19) which for many patients is less than satisfactory. This paper presents a historical scrutiny concerning the views of Rhazes, a pioneer Islamic physician, on enuresis. It begins with a discussion of the diagnosis and treatments of enuresis form Rhazes up now and introduces the reader to nocturnal enuresis according to both modern and traditional Islamic medicine.

## 2. Evidence Acquisition

This is a review study that we searched all available and reliable electronic and paper sources using appropriate keywords about the views of Rhazes in enuresis and compared them with modern medical practice. Rhazes’ Al-Hawi in their original languages (Arabic) was compared with the Persian and English translations to provide a more accurate text. To perform search with appropriate keywords include: “noctural enuresis”, “rhazes”, “Zakaria Rhazes”, “medical education”, “medical history”, “ancient Iran”, “noctural enuresis: diagnosis and treatment” English sources alone or in combination keyword were used in Pubmed, Google-schoolar, Proquest and Medline databases (including Scopus, DOAJ, EBSCO-CINAHL and so on) have been studied in details and the equivalent keyword to search electronic databases containing (Arabic and Islamic data base for example journal of the international society for the history of islamic medicine (JISHIM)), Persian scientific database (SID), Iran university of medical sciences publications database (Iranmedex.ir), Database ministry of health science "Medlib.ir" was searched. Moreover search the Archives Magazines also refer to other electronic resources available in libraries and publications related to Persian Iranian medical scientists, Zakariya Rhazes and also published articles in scientific journals – research on the history of medicine, journal of medical ethics and history of medicine seminars and congresses medical history was made.

We did not focus on the domains of traditional medicine such as the four cardinal humours that were beyond the scope of this paper and we only dealt with items that our current modern medical knowledge obviously and clearly concede with. The literature review was conducted determine the general articles less related opinions of scholars specialized in the field of medical texts and compared with modern medicine. Conceptual framework based on the central research question analyzes the content of each resource and article was selected in relation to the following subject:

1 – Understanding vision of Zakaria Rhazes about types of nocturnal enuresis.

2 – Rhazes administered in nocturnal enuresis.

3 – Comparison of opinion Rhazes and clinical methods with modern medical practice.

In this review we use related studies in the medical literature; particularly recent studies have been applied. The initial search of the medical history and opinion of Rhazes related with keyword mentioned found in 30 – 45 paper related. Moreover search in modern medicine databases about nocturnal enuresis were find more than 800 paper related. Finally near about 10% of articles based on the most similar research objectives and questions were selected. Although care was taken not to deviate from mere scientific discussion, there is undoubtedly probability of errors and mistakes due to the difficult nature of such historical studies. This article can be an opening to comprehensive studies for better exploration of our ancestors’ works.

## 3. Results

Rhazes insight into the concept of noctural enuresis can be perceived from his writings. This great Persian clinician had a profound influence on Arabic as well as European medicine. The two most important books by Rhazes are Kitab al Mansuri and Kitab aI-Hawi. The first one consisted of 10 chapters, including the definition and nature of temperaments and a comprehensive guide to physiognomy. Kitab aI-Hawi was the greatest medical encyclopedia produced by a Moslem physician which was translated into Latin in 1279 and was published in 1486. It was the first clinical text to present the complaints, signs, differential diagnoses and effective treatments of various illnesses. One hundred years later, Avicenna (980 – 1037CE) compiles al-Qanun of Medicine which became a monumental, educational and scientific book with better a classification of different diseases (20). Rhazes was a dedicated observer and while he described the signs and symptoms accurately, he differentiated diverse conditions that produced similar complaints in a methodical and advance way.

Mental health has been identified as an essential component of a person’s general health since the time of the Pharaohs, important enough to place therapy within the divine boundaries of spiritual and religious realms. From the early papyri, through Rhazes, Avicenna, and up to the present day, psychiatry and mental health services have come a long way. The development of knowledge and introduction of pharmacotherapy have not excluded other influences over people’s mental health. Traditional healers continue to play a crucial role as the first source of help for unexplained psychological symptoms that sufferers do not place within the realm of medicine. Culture not only colors the definition of health and disease, but also determines when and where to seek help (21-23). Apart from spiritual treatments, in ancient times urinary problems were also treated with dates, grapes, gum, rush-nuts, wheat, celery, figs, carob, and yellow ochre with varying results (5, 24).

It is evident from Islamic medicine sources that scientific method was implemented as a part of ancient muslims medicine (25). Healing was approached through a combination of scientific, mystical and religious aspects and supernatural influences were considered to be the origin of certain diseases (26). The etiology of enuresis has received considerable attention since the days of antiquity and we find the name of Rhazes associated with it. In order to understand the etiology of enuresis it is well to review briefly the complex mechanism of normal urination. When bladder is filled with urine, increased intravesical pressure is sensed by the bladder wall which sends afferent impulses to the centers of urination in the sacral cord. This stimulus excites the sacral center and results in contraction of the bladder wall and relaxation of the sphincter which results in urination. This is a simple automatic reflex and exists in all infants up to the age of two or three years. After this age the afferent impulses going to the cord also ascend to the cerebrum and depending on the social circumstances the individual can either perform voluntary voiding or inhibit it by contracting the external sphincter. In voluntary urination there is synchronization of muscular action of the bladder which consists of contraction of the bladder muscle and relaxation of the sphincter. Delay in the development of the supra-sacral inhibition mechanisms results in enuresis. Moreover poor nocturnal bladder capacity as well as increased nocturnal urine roduction in the aged people also induces nocturia (27).

The views of Rhazes about some of the causes of bedwetting children are:

1. Relaxation of the muscles located around the bladder outlet.

2. Deep sleep.

3. The mode of stimulation in the urine.

4. Large and heavy food intake before bedtime.

5. Excessive fluid intake before bedtime.

6. Weakness of bladder in holding the urine.

7. A dislocation in the lumbar spine.

8. Excessive cold or hot temperament.

9. Decreased sensitivity to bladder fullness in sleep (28).

In modern medicine the causes of enuresis are described as follows:

1. Delayed development of nervous system and lack of appreciation of bladder fullness.

2. Decreases bladder capacity.

3. Improper antidiuretic hormone cycles which under normal circumstances should rise at night to reduce urine production.

4. Abnormal deep sleep pattern which prevents the child from awakaning when bladder is full.

5. Children suffering from emotional or social stresses may become more prone to enuresis. A very common example is the birth of a new sibling.

6. Children with SNE might have some kind of medical problem, such as urinary tract infection or anxiety (29-31).

In a study performed by Okasha (23) nocturnal enuresis was reported in 1.9% of children. In many families bedwetting was found to be tolerated up to the age of 5 or 6 years and after that, usually between 7 and 10 years, parents decide to do something about it which depends on their tolerance and their social background. The highest number of enuretic children is found in two age groups, 6 to 7 and 11 to 12 years. The apparent rise in the incidence between 6 and 7 years might be due to the anxiety associated with the commencement of school. The rise in 11 to 12 years could be associated with the onset of puberty and the need for independence, widening of the social horizons, relationship with the opposite sex, and self-esteem. At all age groups, Okasha reported that there were more males than females; producing a male/female ratio of 3.2: 1.

About bladder problems one investigation found that while real bladder capacity is identical in children with and without nocturnal enuresis, functional bladder capacity (the volume at which the bladder empties itself) may be less in those with enuresis. No correlation has been found between urethral or meatal stenosis and bed-wetting (5). About emotional or social stresses one study found that 23 to 36 percent of parents had used punishment as their primary means of dealing with bed-wetting. Hence, family education is crucial (11).

Treatment protocols of enuresis described by Rhazes:

1. Refraining from taking any liquids at night.

2. Minimizing the amount of food consumed for dinner.

3. Consuming substances that cause loss of body fluids and substances that can cause urinary retention.

4. Avoiding heavy sleep.

5. Prescription of appropriate medications and certain foods.

6. In refractory cases injecting certain drugs through the urethra into the bladder.

7. Providing special training for conditional learning in patient using psychological approach (28).

Available treatment options for enuresis in modern medicine are:

1. Fluid management (FM) which constitutes a change in the daily drinking pattern with encouragement of drinking fluids during the morning and afternoons and cutting back late evening drinks particularly after dinner and before bedtime. Having an early dinner, and stopping liquid intake from two hours before going to bed are usually recommended.

2. Reward system (RS) in which a “Star Chart” accompanied by little rewards are handed over for dry nights. These rewards are pre-determined by both parents and children before they embarked on the program and the number of dry nights for which a reward is granted increases in a gradual stepwise fashion.

3. Oral or nasal decompression (DDAVP) is the treatment of choice in patients with high urine output at night due to aberrant ADH cycles. The response rate is high but so is the relapse rate after discontinuation.

4. Enuresis alarm therapy remains the most effective and longlasting method to treat enuresis. Intervention with the alarm is associated with a nine times less likelihood of relapse than antidiuretic therapy. Meta-analyses demonstrated that alarm therapy has a 43% lasting cure rate.

5. Anticholinergic drugs in patients with signs of bladder instability should be considered. Side-effects like a dry mouth or flushing are common.

6. The child should be encouraged to empty the bladder at frequent intervals before sleeping.

7. Relieving anxiety and stressful conditions are of utmost importance (25-27).

About oral or nasal desmopressin (DDAVP) for the treatment in patients with high urine output at night a systematic review found that desmopressin reduced the number of wet nights more effectively in children older than nine years and in children who had the fewest number of wet nights. The studies examined in the review found that frequency of wetting decreased anywhere from 10 to 91 percent, but that only 24.5 percent of children achieved complete dryness (14). It seems that many of Rhazes’ comments about the ethiology and treatment of nocturnal enuresis is consistent with modern medical practice. Rhazes also differentiated incontinence in girls and women from enursis and described the causes as follows:

1. High urinary excretion might be associated with excessive thirst, which can be with a sign of “Dyabtys or diabetes.”

2. Prolonged sitting on cold objects or exposure to extreme cold.

3. It can be caused by having a dilute blood.

4. Burning sensation on urination (dysuria) might result in incontinence.

5. Bathing in cold water.

6. Previous urinary bladder damaged or surgery.

7. Bringing out the rock (urinary stone pass).

8. Following a blow or a fall from a height and lumbar spine injury.

He emphasized the importance of the quality of the urine and used to ckeck it for blood, sediments, and thinness (28).

Today in modern medicine finding showed that bedwetting in adult (especially in women and teenager) may be a mixture of reasons that include:

1. Lack the necessary muscle and nerve control or cystocele.

2. Polyuria.

3. Urinary tract infection.

4. Consumption of alcohol, coffee or diuretics.

5. Sleeping pills.

6. Diabetes mellitus or insipidus.

7. Stress and anxiety.

8. Other conditionssuch as neurological problems resulting in neurogenic bladder and sleep apnea can result in bed-wetting (32-35).

In study of Alpha Dian et al.(36) about noctural enuresis in older adults revealed that Nocturia is common in the older adults and is associated with poor sleep, irregular heartbeats, diabetes and stroke.

As can be seen many of the causes mentioned in medical science for incontinence in women and girls were also expressed by Zakaria Rhazes.

## 4. Discussion

A review of opinion Rhazes about nocturnal enuresis reveals that medical practice in those days was comparable to modern medicine yet avoiding the side effects that are commonly experienced with the modern medical approach. Advances in medicine is amongst the most famous and well-known aspects of the Islamic-Iranian civilization, and Iranians flourished profusely in this field. They proved to be superior to their counterparts by holding an experience of six hundred years in training numerous scientist and scholars in this field. Moreover, as an international connection bridge, Iranians had always benefited from the scientific heritage of Romans and Greeks on one hand and from that of India and China on the other hand. In medicine, they mingled the Eastern and Western legacy of medicine and endeavored for its progress and development.

The same medical conditions urologists see in the office today were methodically documented hundreds of years ago. Medical documents clearly illustrate that the ancient Muslims practiced medicine using scientific methods founded on precise clinical observation and physical examination (37). It should be noted that in Islamic countries, autopsy of the human body have always been a controversial subject and despite the reluctance to perform dissection, Iranian physicians could compile a massive body of scientific evidence by just observing the natural history of different patients. New ideas including the heritage of the pre-Islamic civilizations entered the realms of Islamic medicine from the 9th century through systematic translation of foreign text-books such as the writings Galen into Arabic. In the same way as the legacy of their predecessors was studied with great respect, many non-Muslim scientists, particularly Jews and Christians played important roles in the development of the scientific community. The open minded atmospheres in those days, encouraged scientists to engage in debates, share ideas, and seek new knowledge by doing research and examining evidence.
